# A blinded, placebo‐controlled study on the clinical effects of vitamin E supplementation in dogs with osteoarthritis

**DOI:** 10.1111/jvim.16816

**Published:** 2023-07-31

**Authors:** Casey L. Gordon, Samantha J. Reeves, Richard K. Burchell, Craig Thomson, Arnon Gal, Nicolas Lopez‐Villalobos, Natalie S. L. Webster, Kimberley M. Litster, Richard A. S. Mitchell

**Affiliations:** ^1^ Department of Surgery North Coast Veterinary Specialists Sippy Downs Queensland Australia; ^2^ Department of Medicine North Coast Veterinary Specialists Sippy Downs Queensland Australia; ^3^ Department of Veterinary Clinical Medicine University of Illinois Urbana‐Champaign Urbana Illinois USA; ^4^ IVABS Massey University Palmerston North New Zealand; ^5^ Better Veterinary Referrals IDEXX Australia Rydalmere New South Wales Australia

**Keywords:** arthritis, inflammation, lameness, nutraceutical

## Abstract

**Background:**

Vitamin E has a positive effect in the management of osteoarthritis in humans, and in a previous study of dogs. It has been suggested to decrease C‐reactive protein concentrations and liver enzyme activities in humans and animals.

**Objective:**

To assess the effect of vitamin E supplementation on lameness, pain, pain medication requirement, clinical pathology variables, and quality of life in large‐breed dogs with naturally occurring osteoarthritis.

**Animals:**

Fifty‐seven client‐owned dogs with naturally occurring osteoarthritis.

**Methods:**

Dogs received either vitamin E or placebo for 90 days in a randomized, placebo‐controlled, double‐blinded, prospective clinical trial. Clinical lameness scores, pain medication requirements, and owner questionnaires were used to assess response to treatment every 30 days. Blood samples were collected at enrollment and at the end of the study period.

**Results:**

Vitamin E administration did not improve pain, lameness, or quality of life as assessed by owners and veterinarians. Vitamin E supplementation did not decrease the requirement for rescue pain relief. No changes in clinical pathology variables were observed after 90 days of vitamin E supplementation. Body weight was negatively associated with the lameness scores and requirement for rescue pain relief.

**Conclusion:**

Vitamin E supplementation did not have any observable positive effects in dogs with naturally occurring osteoarthritis.

**Abbreviations**
CBPI: canine brief pain inventory
CRP: C‐reactive protein
NSAID: nonsteroidal anti‐inflammatory
OA: osteoarthritis
PIS: pain interference score
PSS: pain severity score
QOL: quality of life
VE: vitamin E

## 1 INTRODUCTION

Osteoarthritis (OA) is a common, multifactorial disease affecting a substantial proportion of pet dogs worldwide.^1^ It can lead to debilitating pain which negatively affects quality of life (QOL), mobility, and behavior, potentially resulting in owner‐elected euthanasia.^2^ Historically, the most common treatment utilized for the management of OA has been nonsteroidal anti‐inflammatory drugs (NSAIDs).^3^ Recent studies suggest that these medications can lead to severe complications, including renal failure and gastric ulceration.^4^ In 1 study, >80% of dogs treated with NSAIDs developed gastric ulceration.^5^ Thus, the adverse effects of these commonly used medications may occur at a higher frequency than previously thought. Therefore, investigation of alternative treatment options, including nutraceuticals, to manage the clinical signs of OA is warranted.^6^, ^7^

The pathogenesis of OA is complex, but damage by synovial free radicals has been identified as a key process. Oxidative stress causes decreased chondrogenesis with subsequent cartilage thinning and is an important aspect in the pathogenesis of OA.^8^ Humans suffering from OA have higher concentrations of oxidants and lower concentrations of antioxidants in synovial fluid, supporting their contribution to the development of OA.^9^ Vitamin E (VE) is a potent antioxidant found naturally in plants, and in vitro testing on animals has elucidated its ability to decrease the effects of oxidative stress on cartilage.^10^, ^11^ It also has been shown to inhibit specific inflammatory pathways associated with OA, as well as to upregulate antioxidants.^12^ Nitric oxide in high concentrations can increase pain signaling, and VE's action of decreasing synovial nitric oxide might decrease clinical signs of pain.^13^ These findings led to research in humans investigating the use of VE supplementation in people suffering from OA. The results showed improvement in both clinical signs and inflammatory markers.^14^, ^15^, ^16^, ^17^, ^18^, ^19^, ^20^, ^21^

The only study investigating the use of VE in dogs was conducted after surgical transection of the cranial cruciate ligament and found that supplementation improved lameness and pain scores.^21^ In addition, it decreased the severity of the cartilage damage observed on necropsy examination and resulted in decreased concentrations of nitric oxide and prostaglandins in the synovial fluid.^21^ These initial results in dogs support the hypothesis that, as in humans, VE might not only help modulate intra‐articular inflammation and cartilage damage but also decrease pain and lameness clinically.

In addition to these postulated benefits in animals and humans with osteoarthritis, VE also may have beneficial effects on the liver and markers of systemic inflammation.^22^, ^23^, ^24^, ^25^, ^26^, ^27^, ^28^ Supplementation has been shown to facilitate management of chronic liver disease in humans.^22^ In a study in dogs, a supplement containing VE was shown to decrease liver enzyme activity and liver size in dogs with and without liver disease.^25^ Another study on dogs with OA showed that although CRP concentrations remained within normal limits, administration of a joint supplement significantly decreased CRP concentrations.^29^ This result was used to support the anti‐inflammatory properties of the supplement. We hypothesized that VE might have similar properties and might decrease CRP concentrations or biochemical markers of liver injury in dogs over a 90‐day study period.

We postulated that VE might be beneficial in dogs with naturally occurring OA and sought to investigate its clinical effects. We hypothesized the VE treatment would decrease lameness and pain scores, decrease pain medication requirements and improve the quality of life (QOL) of dogs with OA. We also hypothesized that VE would decrease C‐reactive protein (CRP) concentrations and liver enzyme activity.

## 2 MATERIALS AND METHODS

### 2.1 Study design

Our study was a randomized, placebo‐controlled, double‐blinded, prospective clinical trial. Dogs were randomized to treatment or control groups using an online randomization tool (https://www.randomizer.org/). All veterinarians, support staff, and owners were blinded as to which group (A or B) was receiving VE and which was receiving placebo. One member of the research team, who was not conducting examinations of dogs, was not blinded to the groups in the event immediate unblinding was necessary. The study was approved by the Community Access Animal Ethics Committee (CA 2021/03/1481).

### 2.2 Enrollment of animals

Client‐owned dogs with clinical and radiographic signs of OA with no other clinically relevant illnesses were recruited.

Dogs of any age, breed, and sex were eligible. To minimize the effect body weight might have on the results, only dogs weighing 20‐40 kg were recruited. Suitable dogs were first assessed by a veterinarian for lameness or joint pain on physical examination that might be consistent with OA. Radiography was then performed to confirm the presence of OA in the affected joints. The radiographs were assessed for OA by a specialist veterinary radiologist. Any dogs with alternative causes of lameness diagnosed on radiographs were excluded from the study.

A CBC and serum biochemistry profile were performed on all dogs after the diagnosis of OA and before enrollment. Dogs were excluded if they were considered systemically ill. This exclusion included any recent illness reported by the owners, any marked changes in the clinical pathology test results suggestive of organ dysfunction, or any concerns on the dog's physical examination, beyond the presence of OA. All dogs were examined by the principal investigator, and the findings were corroborated by either a board‐certified small animal surgeon or a third‐year surgical resident. If dogs were already receiving alternative nutraceuticals (any commercially available joint supplement apart from VE) or pain medication (any veterinary medication prescribed for pain), these factors were recorded. Any concurrent nutraceutical or pain medication had to have been given for at least 3 months before enrollment to ensure no confounding effect was present. Dogs that recently had started new medications or supplements were excluded.

### 2.3 Power sample size analysis

To identify a difference of 1 unit in clinical lameness score between placebo and treatment groups (mean score control group, 18 ± 1; mean score treatment group, 17 ± 1) with a power of 0.95 and alpha error probability of 0.05 the required sample size was estimated to be 27 per group (54 total).^6^ We aimed to recruit 68 dogs (34 per group) to account for an approximately 20% case dropout or loss to follow‐up.

### 2.4 Treatment

Each dog was given 1 dose of either VE powder 400 IU^a^ (White‐E, Virbac Australia Pty Ltd, Milperra, New South Wales) or an equivalent volume of placebo once daily for 90 days. The dose selected was the same given to dogs in a previous VE study, presented in powder form.^21^ In that study, it was found that 400 IU showed a positive effect on pain scores, was within the recommended dose range, and resulted in increased concentrations of VE in synovial fluid and blood samples. The placebo powder was identical to the VE powder except for the active ingredient. Identical scoops were given to each participant to ensure uniform administration. Supplement was measured by weight and the exact quantity of powder was dispensed for each dog on enrollment. Compliance with dosing was assessed by the amount of supplement powder remaining at each review and owner records of administered doses.

### 2.5 Outcome measures

#### 2.5.1 Owner assessment of pain and quality of life

The canine brief pain inventory (CBPI) metric was used to assess owner perception of the dogs' OA and changes noted at home over the course of the study. The questionnaire was completed by owners on days 0, 30, 60, and 90. The CBPI is validated to assess the effects of OA with 3 scores: pain severity score (PIS), pain interference score (PSS), and QOL score.^30^ These were calculated from the values provided by the owners in a questionnaire of 11 questions. The pain severity score is used to assess the degree of pain the owner feels the dog experiences. The pain interference score allows insight into how the pain affects the dog's ability to undertake normal activities. The QOL score is based on the final question and identifies the owner's overall perception of their dog's well‐being.

The owners were unable to see previous scores and were not given any information regarding the veterinarian's assessment when completing the questionnaire. The CBPI was presented to the owners in its validated form for the assessment of dogs with OA.^31^

#### 2.5.2 Veterinarian assessment of pain and lameness

Each dog was assessed for the severity of clinical signs associated with OA at enrollment and at days 30, 60, and 90. The same joint on each dog was assessed at each time point. The lameness assessment was performed by the same veterinarian at each visit. The investigators assessed lameness at a trot, weight‐bearing when standing, pain on palpation, joint range of motion, and assigned an overall severity score. The scoring system yielded a numerical value for each component of the examination, from 1 to 5 (Table A1). The scoring system was adapted from another study assessing supplementation in the treatment of OA.^6^ Body condition scores (BCS; 0‐9) and body weight was recorded at each visit to account for obese animals and any changes in body weight throughout the study.

#### 2.5.3 Rescue pain relief requirement

At enrollment, the owners were advised that pain medications were to be given as necessary to control signs associated with OA, and the number of doses required would be recorded. The pain medication administered to the dogs was 0.1 mg/kg meloxicam (Metacam, Boehringer Ingelheim Animal Health Australia Pty Ltd, North Ryde, New South Wales) PO SID. Owners were advised to record the number of doses given per month. The number of doses was reported to the veterinarian at each visit. Some enrolled dogs already were receiving NSAIDs, or other prescribed pain medications, routinely. These dogs had their initial CBPI and lameness examinations performed with these medications being given as prescribed. This procedure was continued throughout the study to prevent changes in clinical signs or lameness associated with the withdrawal of medication. Additional pain relief was available, at the veterinarian's discretion, if NSAIDs were not suitable or sufficient.

#### 2.5.4 Radiographs

On admission into the study, all affected joints were radiographed for interpretation by a radiologist who was blinded to study group allocation. Each joint was assigned a grade of 0 (no osseous signs of OA), 1 (mild changes), 2 (moderate changes), or 3 (severe changes). Radiography was performed under sedation using 0.002 mg/kg medetomidine (Medetate, Jurox Pty Limited, Rutherford, New South Wales) and 0.2 mg/kg butorphanol (Butorgesic, Troy Laboratories, Glendenning, New South Wales) IV and reversed us 0.01 mg/kg atipamezole (Antipam, Jurox Pty Limited, Rutherford, New South Wales) IM.

#### 2.5.5 Clinical pathology

Blood samples were collected from each dog at enrollment and at day 90. Initial results were used to assess general health before enrollment as well as establish baseline results before VE administration. Results at day 90 were compared to initial results to identify if VE administration had significant effects on clinical pathology results over 3 months.

### 2.6 Statistical analysis

All data analyses were performed using SAS OnDemand for Academics (SAS Institute Inc., Cary, North Carolina). The descriptive statistics of the dependent variables were described by means and SD. The data of the dependent variables were examined for normal distribution by inspection of Q‐Q plots, histogram, and the Shapiro‐Wilk test. Analyses of variance of the dependent variables that had a normal or log‐normal distribution (body weight, rescue remedy, different ordinal scores for the assessment of treatment efficacy) were performed using the MIXED procedure. The models included the fixed effects of treatment, period of the study, interaction between treatment and period, and body weight as a covariate. The model also included the dog as a random effect to account for repeated measures on the same dog over time. The marginal mean and standard errors of each of the dependent variables for treatment, period, and the interaction between treatment and period were obtained and used for multiple mean comparisons with the Fisher's least significant difference test for post hoc pairwise comparisons as implemented in the LSMEANS option of the MIXED procedure.

For variables that did not have a log‐normal distribution and instead had a skewed (Poisson) distribution, analyses of variance were performed with the GLIMMIX procedure by a log link transformation with a model that included the same fixed and random effects as described for the above variables. Multiple mean comparisons were performed on the log scale, but results are presented as back‐transformed marginal means and standard errors.

Analyses of variance of hematologic and biochemical variables between treatments at the beginning and the end of the study also were performed using the MIXED procedure (for variables that followed a normal or log‐normal distribution) or the GLIMMIX procedure (for those with a Poisson distribution) with a log link transformation. The model included the fixed effects of treatment and the baseline value of the dependent variables, and dog as a random effect to account for repeated measures on the same dog over time.

The Chi‐squared test as implemented in the FREQ procedure was used to determine if differences were present between treatment and control groups with respect to the proportion of dog breeds, dogs receiving nutritional supplements, distribution of BCS across groups or with respect to the proportion of arthritic joints. The difference in BCS between groups was analyzed using the Mann‐Whitney *U* test. Significant effects were concluded if *P* < .05.

## 3 RESULTS

### 3.1 Participants

**FIGURE 1:**
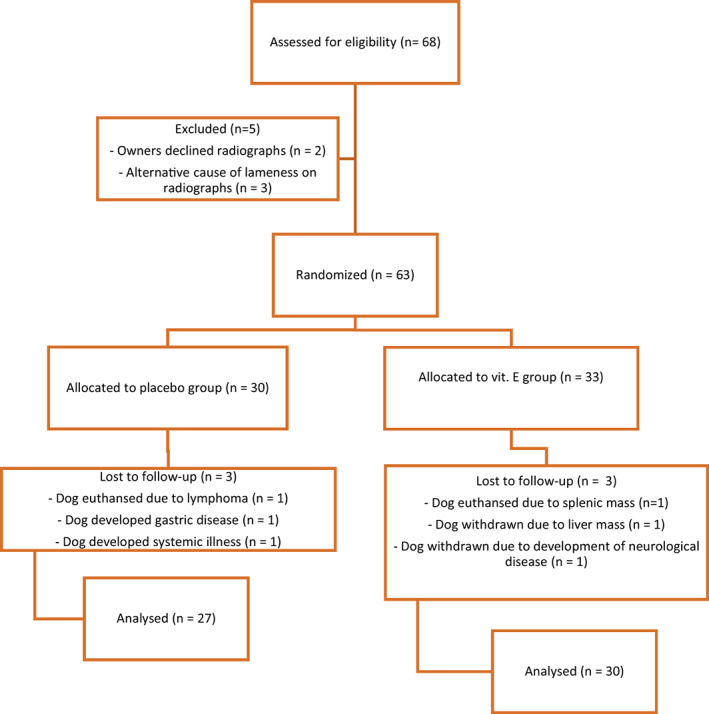
CONSORT flow diagram of dogs with osteoarthritis that were randomized to receive treatment with placebo or vitamin E over 3 months

Sixty‐eight dogs were assessed for eligibility and 5 dogs were excluded (3 dogs had alternate causes of lameness and 2 dogs were withdrawn by the owners; Figure 1). Fifty‐seven of the 63 dogs completed the study, but not all data were available for all dogs (1 dog was euthanized during the study for splenic mass rupture confirmed on necropsy, 1 dog was withdrawn from the study because of unwillingness to ingest supplement powder, 2 dogs were withdrawn because of development of systemic illness, 1 dog was withdrawn because of lymphoma, and 1 dog was withdrawn because of liver neoplasia).

Thirty dogs were randomly allocated to receive VE and 27 dogs received placebo. The mean ± SD age (years) of the dogs in the VE group (9.5 ± 3.2) did not differ from the placebo group (10.8 ± 2.8; *P* = .09). The mean ± SD body weight (kg) of dogs in the VE group (33 ± 6) was significantly higher than in the placebo group (28 ± 6; *P* < .001), but median BCS did not differ between groups (6; *P* = .38). No significant changes in mean (±SE) body weight (kg) within and between groups were found over the course of the study (VE group end of first month, 29.8 ± 0.3; end of second month, 29.7 ± 0.4; end of third month, 29.7 ± 0.3; placebo group end of first month 30.0 ± 0.3; end of 2nd month 30.1 ± 0.3; end of third month 29.9 ± 0.3; *P* = .83).

Elbows (55 joints, 34.4% of all joints) and hips (66 joints, 41.3% of all joints) were significantly more affected by OA than other joints (*P* < .001) but, dogs enrolled in the study also had OA in the carpi (n = 7; 4.4%), tarsi (n = 2; 1.3%), stifles (n = 17; 10.6%), shoulders (n = 1; 0.6%), and lumbosacral spine (n = 12; 7.5%). Both groups had a similar distribution of affected joints with no joints significantly over‐represented in 1 group (*P* = .93). A similar number of affected joints was observed in dogs of both groups (*P* = .93).

Radiographs were performed on 134 joints and the results of the radiologist scoring is presented in Table 1. No significant difference was found in the distribution of OA grades across the groups (*P* = .39) or in the marginal mean (±SE) of OA grades between groups (VE 1.149 ± 0.149; placebo, 1.461 ± 0.199; *P* = .21).

**TABLE 1:** Radiographic osteoarthritis grades of dogs treated with placebo or VE for 3 months

OA Grade	0	1	2	3	Total
Placebo	11 (20%)	19 (34%)	11 (20%)	15 (26%)	56
Vitamin E	20 (26%)	33 (42%)	12 (15%)	13 (17%)	78

*Note*: 0—no radiographic evidence of OA, 1—mild radiographic evidence of OA, 2—moderate radiographic evidence of OA, and 3—severe radiographic evidence of OA.

### 3.2 Clinical lameness and owner questionnaire findings

The CBPI consists of 3 scores to assess the clinical severity of OA in dogs: pain severity score (PSS), pain interference score (PIS), and the QOL score. The marginal mean (±SE) of the PSS for dogs in the VE group was 12.7 ± 1.4. It did not differ from PSS in the placebo group (14.3 ± 1.5; *P* = .45). In both groups, PSS significantly decreased over time (*P* = .02). The marginal mean (±SE) of the PIS in the VE group (21.3 ± 2.4) did not differ from the placebo group (25.3 ± 2.6; *P* = .27). In both groups, PIS significantly decreased over time (*P* < .001). The marginal mean (±SE) of the QOL score of dogs in the VE group (3.6 ± 0.2) did not differ from QOL in the placebo group (3.5 ± 0.2; *P* = .63). However, in the VE group, the marginal mean (±SE) QOL significantly increased over time in the first 60 days (day 0, 3.27 ± 0.17; day 30, 3.75 ± 0.20; day 60, 3.82 ± 0.21; *P* < .05). The placebo group had no change in the marginal mean (±SE) QOL over the same time (day 0:3.34 ± 0.18, day 30:3.39 ± 0.21, day 60:3.51 ± 0.21, *P* < .05).

No difference was found between the treatment groups in any aspect of the clinical lameness assessment. The scores in both groups improved over the 90 days. Results of the lameness scoring are presented in Table 2.

**TABLE 2:** Clinical Lameness Scores (marginal mean ± SE) of placebo and VE dog groups

Score/question	Placebo				Vitamin E			
	Day 0	Day 0	Day 30	Day 60	Day 90	Day 30	Day 60	Day 90
Lameness	2.59 ± 0.15	2.18 ± 0.13	1.70 ± 0.15	1.66 ± 0.16	1.60 ± 0.15	1.96 ± 0.16	1.93 ± 0.16	1.84 ± 0.16
Pain on palpation	3.35 ± 0.14	3.37 ± 0.13	2.96 ± 0.16	2.53 ± 0.17	2.43 ± 0.16	2.70 ± 0.16	2.59 ± 0.16	2.64 ± 0.16
Joint mobility	3.31 ± 0.24	2.79 ± 0.22	2.55 ± 0.24	2.77 ± 0.25	2.30 ± 0.24	2.79 ± 0.25	2.49 ± 0.25	2.67 ± 0.25
Weightbearing	2.25 ± 0.12	2.06 ± 0.11	1.62 ± 0.13	1.56 ± 0.14	1.65 ± 0.14	1.87 ± 0.13	1.82 ± 0.14	1.71 ± 0.14
Overall	2.90 ± 0.32	2.51 ± 0.28	2.26 ± 0.33	2.22 ± 0.37	2.12 ± 0.33	2.55 ± 0.35	2.38 ± 0.36	2.18 ± 0.33

Body weight was analyzed as a confounding factor in all lameness and CBPI scores. Increasing body weight had a marginal effect on the lameness score as assessed by the veterinarian (*P* = .05) and a significant effect on the dog's ability to rise from lying as assessed by the owner (*P* = .05).

### 3.3 Pain medication requirement findings

The administration of additional nutraceuticals did not significantly differ between groups (VE 51.7%; vs placebo 48.3%; *P* = .35), and no significant differences were found in the percentage of dogs requiring at least 1 dose of rescue pain medication (VE, 32%; placebo, 25%; *P* = .62). Treatment (ie, VE vs placebo) did not have a significant effect on the marginal mean (±SE) number of times rescue pain medication was administered to either the VE group (7 ± 3) or the placebo group (9 ± 3; *P* = .78), but significant effects of time (*P* = .01) and the interaction between treatment and time (*P* = .003) in the VE group were found. The VE group had increased use of pain medication at days 0, 30, and 60 with a decrease in use at day 90 (respective marginal mean [±SE] number of doses: 6 ± 3, 7 ± 3, 9 ± 3, 7 ± 3) whereas the placebo group had no change in pain medication requirement across the study period. Increased body weight also resulted in more frequent requirement of rescue pain medication (*P* = .04).

### 3.4 Clinical pathology findings

Three months of VE administration did not lead to significant differences in serum biochemical variables at the end of the study compared to baseline. The mean (±SD) CRP concentration was within normal limits in the VE group (5.02 ± 10.59) and the placebo group (3.62 ± 5.21) and no change in CRP concentration was found in either group from day 0 to 90 (*P* = .95).

## 4 DISCUSSION

Overall, we showed that VE administration did not improve outcomes in dogs with OA, when compared to placebo. In contrast to the previous study in dogs and many studies in humans, VE supplementation had no positive effect on QOL, pain, lameness, or clinical pathology variables. Also, no decrease in pain medication requirement was observed.

The results of the CBPI showed that administration of VE improved QOL in dogs with OA over the first 60 days, but, when compared to the placebo group, no significant difference was identified. It did not have a positive effect on pain, lameness, or function. Poor QOL is the leading cause of owner‐elected euthanasia in dogs.^32^ For this reason, it also has been suggested that the success of a new treatment could be defined by an improvement in QOL as reported by the owners.^33^ These results show that VE supplementation did not improve the QOL in dogs when compared with placebo, based on owner assessment, and therefore cannot be considered an appropriate treatment option in OA.

Our study did not identify an improvement in pain or lameness in dogs treated with VE as evaluated by veterinarians. These findings differ from the results of studies in humans suggesting that VE can decrease the pain associated with naturally occurring OA. Our findings also differ from the results obtained in the previous veterinary study where VE supplementation decreased pain and lameness after surgical transection of the cranial cruciate ligament in dogs. Disparities in these results might be because the dogs in the previous study were experiencing acute joint inflammation without the onset of chronic changes associated with advanced OA. Investigation of VE supplementation in dogs with acute joint inflammation, such as those undergoing arthroscopy, arthrotomy, or acute septic or aseptic arthritis might be warranted. It is also possible that the formulation of VE used was not successful at increasing in vivo concentrations, and bioavailability studies would be ideal. A study performed on dogs showed that VE concentrations in synovial fluid of dogs with OA were increased, rather than decreased as was the case in previous studies in humans.^34^ These findings call into the question the function of VE in joints. We considered whether increased VE was associated with increased mobilization to synovial fluid to offer chondroprotective effects. It is also possible however that there is minimal utilization of VE by the synovium. Further investigation into the movement and utilization of VE in inflammatory joint disease in dogs is warranted.

It is also possible that a numerical scoring system could not detect subtle changes in gait and lameness. Although regularly utilized in both clinical and research settings of veterinary medicine, numerical lameness scoring is not the most sensitive method to detect changes in gait and does not correlate well with objective measurements such as force plate analysis.^6^, ^35^ The same blinded observer was used throughout the study to minimize the effect the subjective nature of the scoring system would have on results. In agreement with the veterinary assessment, the CBPI scores for pain and lameness, as assessed by the owners, did not improve after VE supplementation. The CBPI has been validated to assess owners' perception of pain, loss of function, and QOL of dogs suffering from OA, but it does not correlate well with force plate analysis.^31^ An objective assessment of lameness after VE supplementation is warranted to detect more subtle improvements in gait.

Pain relief has been a mainstay of treatment for dogs with OA.^2^, ^3^ The most used pain medication, NSAIDs, recently have been shown to have more frequent adverse effects than those that are clinically apparent.^4^, ^5^ Therefore, it would be beneficial to find safer alternatives to minimize the use of these medications. We postulated that if VE has positive effects on the pain and lameness associated with OA, it may decrease the requirement for other medications.

C‐reactive protein is an acute inflammatory marker in dogs that is not affected by OA.^36^ A recent study found that although the results in dogs with OA remained normal, administration of a PO joint supplement to dogs resulted in a further decrease in CRP concentrations, supporting its anti‐inflammatory properties.^29^ Vitamin E has anti‐oxidant and proposed anti‐inflammatory effects in the joints, which may have a similar effect in further decreasing CRP. Our results found no increase in CRP concentrations in dogs with OA, consistent with previous findings, but no further decrease in CRP concentrations was found after VE administration. This result supports previous findings that CRP has no diagnostic value in assessing the presence or severity of OA in dogs, and that VE does not decrease CRP concentrations. Recently, serum CRP concentration and its correlation with clinical signs of OA in humans have been discussed when high‐sensitivity testing is performed.^36^, ^37^ Further evaluation of high‐sensitivity CRP in dogs may be warranted.

Body weight had a significant confounding effect on several outcome measures in our study. Increased body weight resulted in higher veterinarian‐assessed lameness scores, lower ability to rise scores by owners, and increased use of rescue pain medication. This finding supports the results of other studies suggesting that OA often causes more severe clinical signs in larger or heavier dogs.^2^, ^38^ In these studies, it was not clear whether the increased weight of the affected dogs was associated with breed or body condition. In 1 study, obesity was found to quadruple the risk of cranial cruciate disease in dogs.^39^ In our study, it was breed‐related weight that affected the outcomes, rather than increased weight associated with obesity. In our study, even though allocation of dogs was randomized and the weight range was limited, the dogs in the VE group were significantly heavier than those in the placebo group. This disparity might have resulted in more severe clinical signs in the treatment group and could have contributed to the lack of improvement in lameness and pain.

Our study had several limitations. The use of a numerical lameness scoring in dogs has lower sensitivity than force plate analysis or other objective gait analysis techniques. These techniques may have been able to detect more subtle changes in gait. The lack of availability of force plate analysis in private practice limits the use of this technology. Another limitation is the difference in body weight between the 2 groups. Although attempts were made to prevent this difference by limiting the weight of dogs enrolled, the use of matched controls rather than random allocation might have been warranted. The PO supplement utilized was a unique formulation not used in previous studies. This formulation was the only registered and suitable product available, but no bioavailability studies were available for the product. It would have been ideal to test blood or synovial fluid to ensure that in vivo results were appropriately increased after administration, but doing so was not possible because of cost limitations.

Further investigation into the use of VE for dogs with OA is warranted. Such studies could include objective lameness assessment, such as kinematic or force plate analysis, to identify subtle changes. Investigation into the use of VE in more acute inflammatory joint conditions also is warranted. Confirming the bioavailability of the product is recommended in future studies.

## 5 CONCLUSION

Our findings show that administration of VE at 400 IU per day to dogs with naturally occurring OA did not improve QOL, lameness, pain, biochemical variables, or pain relief requirements when compared to placebo. The use of VE supplements in dogs affected by OA cannot be recommended based on our results.

## CONFLICT OF INTEREST DECLARATION

Authors declare no conflict of interest.

## OFF‐LABEL ANTIMICROBIAL DECLARATION

Authors declare no off‐label use of antimicrobials.

## INSTITUTIONAL ANIMAL CARE AND USE COMMITTEE (IACUC) OR OTHER APPROVAL DECLARATION

Approved by the Community Access AEC, Ecosciences Precinct, 41 Boggo Road, Dutton Park, QLD, 4102, approval number CA 2021/03/1481.

## HUMAN ETHICS APPROVAL DECLARATION

Authors declare human ethics approval was not needed for this study.

## Appendix

**TABLE A1:** Marginal mean (±SE) canine brief pain inventory (CBPI) scores of dogs with osteoarthritis treated with placebo or vitamin E over 3 months

Score/question	Vitamin E				Placebo				TX	T	TX * T	BW
	T1	T2	T3	T4	T1	T2	T3	T4	*P*	*P*	*P*	*P*
Q1	3.37 ± 0.13	2.96 ± 0.16	2.53 ± 0.17	2.43 ± 0.16	3.35 ± 0.14	2.70 ± 0.16	2.59 ± 0.16	2.64 ± 0.16	.96	<.0001	.3664	.8429
Q2	1.78 ± 0.35	1.76 ± 0.41	1.52 ± 0.40	1.99 ± 0.47	2.17 ± 0.45	1.90 ± 0.43	2.14 ± 0.49	1.46 ± 0.36	.78	.79	.254	.277
Q3	3.87 ± 0.38	3.31 ± 0.42	2.68 ± 0.45	3.13 ± 0.43	4.00 ± 0.41	3.65 ± 0.44	3.70 ± 0.45	3.16 ± 0.44	.48	.0037	.2675	.4447
Q4	2.85 ± 0.47	2.13 ± 0.44	1.98 ± 0.46	2.56 ± 0.52	3.19 ± 0.56	3.11 ± 0.59	2.90 ± 0.57	2.24 ± 0.46	.42	.1745	.2166	.6961
Q5	3.88 ± 0.51	2.37 ± 0.43	2.18 ± 0.44	2.86 ± 0.51	4.17 ± 0.59	3.69 ± 0.58	3.52 ± 0.58	3.47 ± 0.57	.11	.0036	.2179	.5461
Q6	3.23 ± 0.49	1.80 ± 0.37	1.59 ± 0.37	2.70 ± 0.52	4.13 ± 0.66	3.39 ± 0.60	3.62 ± 0.65	2.81 ± 0.52	.04	.0007	.0224	.9684
Q7	5.81 ± 0.48	4.17 ± 0.56	3.18 ± 0.61	3.92 ± 0.57	4.94 ± 0.52	4.26 ± 0.57	4.05 ± 0.59	3.67 ± 0.57	.95	<.0001	.1835	.0467
Q8	3.88 ± 0.48	2.69 ± 0.55	2.62 ± 0.60	2.93 ± 0.56	4.59 ± 0.52	4.01 ± 0.59	3.45 ± 0.58	3.10 ± 0.57	.24	.0018	.5402	.7985
Q9	4.99 ± 0.54	3.37 ± 0.61	2.99 ± 0.66	4.12 ± 0.62	5.82 ± 0.58	4.60 ± 0.65	4.36 ± 0.64	4.05 ± 0.63	.26	<.0001	.3018	.9321
Q10	5.69 ± 0.52	3.57 ± 0.60	3.36 ± 0.65	3.78 ± 0.61	5.91 ± 0.56	4.98 ± 0.64	4.36 ± 0.63	4.68 ± 0.62	.21	<.0001	.4755	.3568
Q11	3.27 ± 0.17	3.75 ± 0.20	3.82 ± 0.21	3.47 ± 0.20	3.34 ± 0.18	3.39 ± 0.21	3.51 ± 0.21	3.64 ± 0.20	.63	.0403	.1399	.1093

Abbreviations: BW, bodyweight; Tx, treatment; T, time; Tx × T, interaction between treatment and time; *P* < .05 is considered significant.
